# Nrf3: an emerging player in cancer, inflammation, and cellular homeostasis

**DOI:** 10.1007/s11033-026-11678-1

**Published:** 2026-03-16

**Authors:** Deniz Kilicaslan, Meliha Burcu Irmak-Yazicioglu, Erna Copuroglu

**Affiliations:** 1 Department of Bioengineering, Faculty of Chemical and Metallurgical Engineering, Yıldız Technical University, Istanbul, Turkey; 2 Department of Molecular Biology and Genetics, Faculty of Science and Literature, Haliç University, Istanbul, Turkey; 3 Graduate Program of Molecular Biology and Genetics, Institute of Graduate Studies, Haliç University, Istanbul, Turkey

**Keywords:** Nrf3, Cap’n Collar, Cancer, Antioxidant

## Abstract

Nuclear factor erythroid 2-related factor 3 (Nrf3) is a transcription factor of the CNC-bZIP family that plays a critical role in managing cellular stress, redox balance, and internal homeostasis. Initially localized to the endoplasmic reticulum, Nrf3 undergoes a sophisticated activation process to reach the nucleus and regulate gene expression. Emerging evidence identifies Nrf3 as a multifaceted and context-dependent regulator whose biological impact varies significantly across different tissues and disease states. In cancer biology, Nrf3 exhibits a dual role by acting as either an oncogene or a tumor suppressor. In many malignancies, such as colorectal and pancreatic cancers, it promotes tumor growth by driving cell cycle progression, metabolic adaptation, and cancer cell survival. Conversely, in other contexts like breast cancer, Nrf3 can inhibit tumor progression by increasing stress signals and suppressing pathways that lead to cell migration. Beyond its role in oncology, Nrf3 coordinates responses to inflammation and metabolic shifts, often under the control of various cellular signals and microRNAs. This review highlights its importance in maintaining cellular health and underscores its potential as a clinical biomarker and therapeutic target, while calling for further research to fully clarify its diverse biological functions.

## Introduction

### Domain architecture of Nrf3

Basic leucine zipper (bZIP) transcription factors represent a large family of proteins that are broadly conserved among eukaryotic organisms, characterized by the presence of an α-helical bZIP dimerization domain and a DNA-binding domain [1]. These factors control the transcriptional activity of numerous genes that play critical roles in the regulation of various cellular processes by binding to DNA as homodimers or heterodimers [2, 3]. Within this large family, the Cap’n Collar (CNC) subfamily is distinguished by the presence of a conserved CNC domain in addition to the bZIP motif. NFE2 (p45-NFE2) is a prominent member of the CNC family of transcription factors, which also includes NFE2-related factors (NFE2L1, NFE2L2, and NFE2L3). Bach1 and Bach2 proteins, although structurally related, are functionally distinct CNC-type bZIP transcription factors. CNC proteins heterodimerize with small Maf (sMaf) proto-oncogenes to bind Maf recognition elements (MARE) in DNA, thereby enabling the transcriptional activation or repression of various target genes [4, 5].

CNC transcription factors orchestrate fundamental biological processes, including the regulation of gene expression, organismal development, tissue differentiation, and the maintenance of cellular homeostasis [6–8]. Among these factors, NFE2L2 (Nrf2) is recognized as a principal cellular defender that becomes activated in response to oxidative stress and has emerged as the most extensively investigated CNC member, particularly in relation to cancer and various other pathologies [9–11]. Nrf2 predominantly localizes in the cytoplasm, where it is sequestered by Kelch-like ECH-associated protein 1 (KEAP1). In contrast, NFE2L1 (Nrf1) and NFE2L3 (Nrf3) possess an N-terminal domain (NTD) and are anchored to the endoplasmic reticulum (ER) membrane. Notably, Nrf1 regulates the transcription of proteasome subunit genes, thereby contributing to protein quality control and degradation [12]. However, the precise physiological functions of Nrf3 -the most recently identified member of the CNC family- particularly in cancer progression, antioxidant defense, and apoptosis, remain poorly elucidated.

**Fig. 1:**
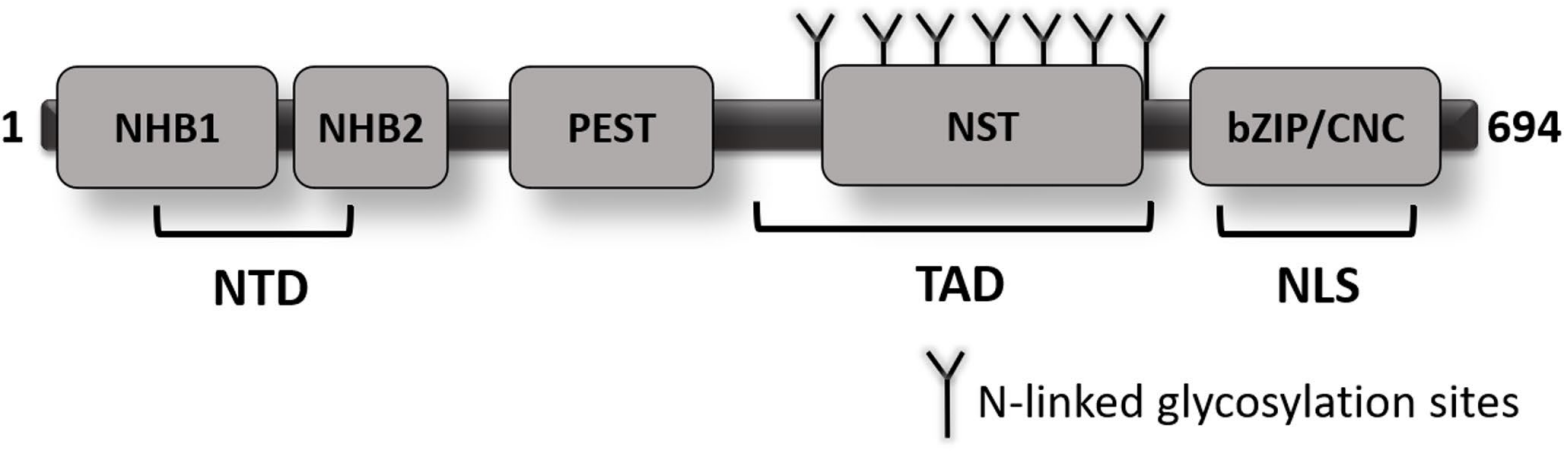
Schematic representation of the human Nrf3 protein structure. The protein consists of several conserved domains, including the N-terminal domain (NTD), which contains the N-terminal homology box 1 (NHB1) and box 2 (NHB2). A PEST sequence-rich in proline (P), glutamic acid (E), serine (S), and threonine (T)-is also present and may contribute to protein turnover. The transactivation domain (TAD) encompasses the NST region, which contains multiple predicted N-linked glycosylation sites. The C-terminal region harbors the basic leucine Zipper/Cap’n’Collar (bZIP/CNC) domain, which is responsible for DNA binding and dimerization, and includes a nuclear localization signal (NLS)

Nrf3 has been suggested to exist in three distinct forms: form A localized to the ER, form B present in the cytoplasm, and form C predominantly located in the nucleus [13]. Structurally, Nrf3 harbors a conserved NTD (Fig. 1), within which the N-terminal homology box 1 (NHB1) signal sequence mediates its targeting to the ER membrane [14]. Analogous to Nrf1, the ER-associated form A of Nrf3 undergoes N-glycosylation as a post-translational modification. Following this modification, Nrf1 is translocated from the ER lumen into the cytosol, where it undergoes deglycosylation and proteolytic processing prior to its nuclear import. The cleavage of Nrf1 by the aspartic protease DNA damage-inducible 1 homolog 2 (DDI2) is a critical step in facilitating the transcriptional activation of proteasome subunit genes [15–17]. Importantly, DDI2 is also required for the nuclear translocation of Nrf3, as siRNA-mediated knockdown of DDI2 significantly impairs this process [18].

Stimulation of cells with tumor necrosis factor-alpha (TNF-α) or arginine leads to the upregulation of Nrf3 expression; the underlying mechanism of this increase is attributed to glycosylation, a post-translational modification [4, 13, 19, 20]. This observation raises the possibility that, similar to Nrf1, Nrf3 may require deglycosylation as a prerequisite for its translocation into the nucleus. Nonetheless, the precise functional role of the N-terminal homology box 2 (NHB2) motif located within the NTD of Nrf3 has yet to be elucidated.

### ERAD-mediated regulation of Nrf3

**Fig. 2:**
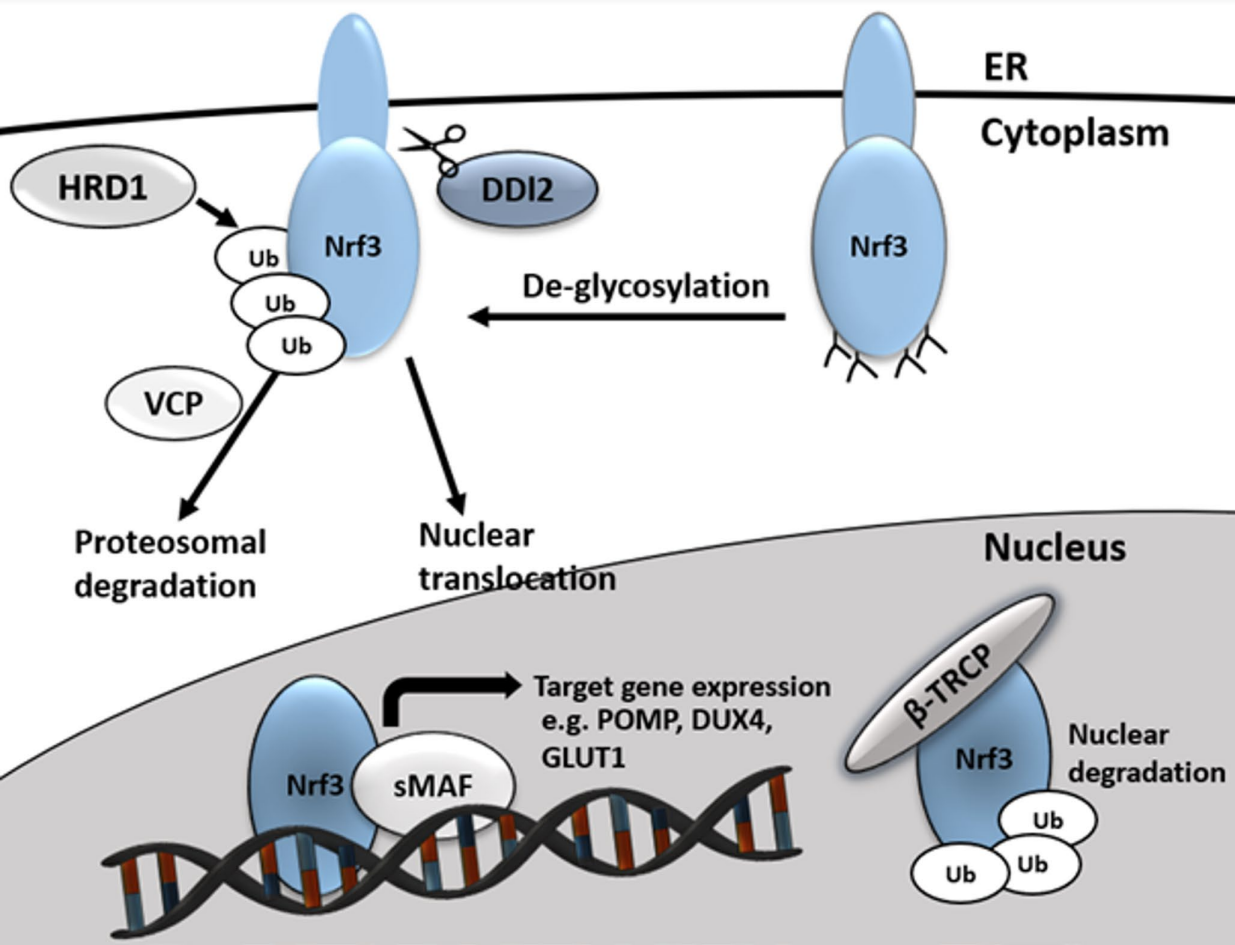
Schematic representation of Nrf3 processing and regulation. At the cytoplasmic level, Nrf3 is targeted for proteasomal degradation via polyubiquitination by the HRD1/VCP complex; conversely, its active form is liberated through proteolytic cleavage mediated by the DDI2 protease. Upon nuclear translocation, Nrf3 heterodimerizes with sMAF proteins to induce the transcription of target genes, such as POMP and GLUT1, while the nuclear signaling phase is terminated through β-TRCP-mediated degradation.

The cytoplasmic regulation of Nrf3 is primarily governed by the endoplasmic reticulum-associated degradation (ERAD) pathway (Fig. 2). ERAD facilitates the retrotranslocation of misfolded Nrf3 proteins from the ER lumen into the cytosol, where they undergo polyubiquitination and are subsequently degraded by the 26 S proteasome [21]. Within this pathway, Nrf3 is polyubiquitinated by the E3 ubiquitin ligase HRD1, recognized by the AAA-ATPase p97/valosin-containing protein (VCP), and targeted for proteasomal degradation [18]. In addition, Nrf3 stability is regulated by the Skp1-Cullin1-F-box (SCF) ubiquitin ligase complex via the F-box protein FBW7 and glycogen synthase kinase 3 (GSK3), which phosphorylates Nrf3, promoting its polyubiquitination by FBW7 [22]. In the nucleus, Nrf3 is further degraded by β-TRCP, another substrate-recognizing component of the SCF complex [18].

In the ER, the inactive form of Nrf1 is known to undergo deglycosylation by N-glycanase 1 (NGLY1) [23]. However, no direct evidence currently supports a similar deglycosylation process for Nrf3. Although the regulatory mechanisms of Nrf3 share several parallels with those of Nrf1, particularly in terms of ER processing and proteasomal degradation, further investigation is required to delineate the precise activation mechanisms of Nrf3.

### Physiological roles of Nrf3

Despite increasing interest in Nrf3, its physiological roles remain largely undefined, and in vivo data are limited. In contrast to the ubiquitous expression patterns of Nrf1 and Nrf2, Nrf3 is predominantly expressed in the placenta, with only low-level expression detected in most other tissues [24]. Although Nrf3-knockout mice do not exhibit overt developmental or morphological abnormalities [25], exposure to the potent carcinogen benzo[a]pyrene results in the development of thymic lymphomas and metastatic lung tumors in these animals [24, 26]. These findings suggest that, while Nrf3 deficiency does not impair normal development, it may predispose organisms to chemical carcinogenesis and tumor progression under stress conditions.

Beyond its roles in cancer, Nrf3 has also been shown to play a functional role in smooth muscle cell (SMC) differentiation. During SMC differentiation, Nrf3 expression levels increase; moreover, its silencing via siRNA has been reported to impair the expression of key SMC markers such as smooth muscle α-actin and the transcription factor myocardin [27]. Moreover, overexpression of Nrf3 has been reported to significantly increase reactive oxygen species (ROS) levels in breast cancer cells [28]. Studies conducted in embryonic stem cells have demonstrated that Nrf3 suppresses the expression of antioxidant enzymes such as NAD(P)H quinone dehydrogenase 1 (NQO1) and peroxiredoxin (Prdx), while promoting ROS generation through the upregulation of NADPH oxidase 4 (Nox4), a major intracellular source of ROS [27]. Given the pivotal role of ROS in regulating stem cell differentiation, it has been proposed that Nrf3-mediated ROS accumulation may function as a critical regulatory factor in cellular differentiation and fate determination processes.

Nrf3 expression is also sensitive to ER stress. The ER serves as a central hub for protein folding, synthesis, and redox regulation [29]. Disruption of ER function leads to the accumulation of misfolded proteins and the activation of the unfolded protein response. Experimental induction of ER stress in COS-1 cells using tunicamycin, an inhibitor of N-linked glycosylation, or brefeldin A, which impairs ER-to-Golgi protein trafficking, has been shown to markedly upregulate Nrf3 expression [14]. Similarly, treatment of embryonic stem cells with thapsigargin, a sarco/endoplasmic reticulum Ca²⁺-ATPase inhibitor, also resulted in a pronounced upregulation of Nrf3 [27]. These findings further support the role of Nrf3 as a stress-responsive transcription factor regulated by ER homeostasis. In light of its structural intricacy and complex regulatory mechanisms, an increasing volume of research has begun to uncover the context-dependent functions of Nrf3 across a spectrum of pathophysiological conditions.

## Functional roles of Nrf3 in inflammation, cancer, proteasome dynamics and redox biology

Nrf3 has emerged as a context-dependent regulator that interfaces immune signaling, malignant transformation, proteasome homeostasis, and redox balance. In the following sections, we delineate how Nrf3 modulates pro-inflammatory cytokine circuits, orchestrates oncogenic and tumor-suppressive pathways in a tissue-specific manner, coordinates proteasome gene expression in concert with Nrf1/Nrf2, and fine-tunes cellular redox status by interacting with ARE-driven antioxidant networks. Highlighting these functional axes provides an integrated view of Nrf3 as a multifaceted transcription factor with far-reaching implications for pathophysiology and therapy.

### Nrf3 and inflammation

Inflammation is a highly orchestrated and multifaceted biological response to tissue injury induced by various stimuli, including physical trauma, pathogenic infections, and exposure to toxic agents. This process is characterized by the activation of immune cells and the augmented release of pro-inflammatory cytokines within the inflammatory microenvironment. Kobayashi et al. demonstrated that Nrf3 is expressed not only in placental tissue but also in cell lines derived from multiple myeloma and Burkitt’s lymphoma. Furthermore, while Nrf3 expression is undetectable in pre-B cells, it is reportedly upregulated in mature B cells and cells of the monocyte lineage [30]. In a study investigating the role of Nrf3 in adaptive immunity, Nrf3-knockout (Nrf3⁻/⁻) mice infected with lymphocytic choriomeningitis virus exhibited antigen-specific CD4⁺ and CD8⁺ T cell responses, as well as B cell responses, that were comparable to those observed in wild-type counterparts [25].

Nrf3 may also contribute to the regulation of tissue repair and wound healing. Nrf3 expression is upregulated in Nrf2⁻/⁻ mice, suggesting a potential compensatory response in the absence of Nrf2. Furthermore, keratinocyte growth factor (KGF) has been reported to enhance Nrf3 expression in keratinocytes [31]. In ultraviolet (UV)-irradiated skin of Nrf3⁻/⁻ mice, neutrophil infiltration and the expression levels of pro-inflammatory cytokines interleukin-1β (IL-1β) and interleukin-6 (IL-6) were found to be reduced compared to wild-type controls [32].

The adaptation of cancer cells to the hypoxic microenvironment directly influences not only cellular survival pathways but also the mechanisms of tumor immune evasion; indeed, it has been reported that therapeutic stress applied under hypoxic conditions can contribute to tumor aggressiveness by altering the balance between apoptosis and autophagy [33]. In this context, immune cells and cytokines within the tumor microenvironment play a pivotal role in maintaining chronic inflammation and promoting tumor progression. Furthermore, Nrf3 has been identified as a gene associated with decreased immune cell infiltration, particularly in patients with kidney renal clear cell carcinoma, which exhibits high hypoxic characteristics [34]. This finding suggests that Nrf3 may contribute to the suppression of immune responses within the tumor milieu. On the other hand, pro-inflammatory cytokines such as TNF-α have been shown to exert regulatory effects on Nrf3 expression. Indeed, TNF-α has been reported to upregulate Nrf3 expression at both mRNA and protein levels in JAR choriocarcinoma cells [4]. TNF-α exerts its biological effects primarily through the activation of various signaling pathways, including nuclear factor kappa B (NF-κB) and JNK [35]. In colon cancer cell lines, TNF-α treatment enhances the expression of RELA, one of the five core subunits of the NF-κB complex, which in turn binds to the first intron of the Nrf3 gene and positively regulates its transcriptional activity [19].

Additionally, in colon cancer cells, Nrf3 is transcriptionally induced by the β-catenin/TCF4 complex via binding to the wnt responsive element (WRE) sequence located within the first two introns of the gene [36]. Beyond β-catenin, interferon-gamma (IFN-γ) has also been reported to increase Nrf3 expression [37]. These findings suggest that pro-inflammatory cytokines such as TNF-α and IFN-γ contribute to the regulation of Nrf3, highlighting its potential involvement in immune system modulation.

### Nrf3 and cancer

Cancer development is strongly influenced by transcription factors that regulate cell cycle progression, apoptosis, and cellular stress responses. Among these, members of the CNC-bZIP family have been extensively studied for their roles in tumorigenesis, with Nrf2 being a well-established oncogenic driver in several cancers [38–40]. In contrast, Nrf3 remains less characterized; however, growing evidence suggests that its dysregulated expression contributes to malignant transformation, tumor growth, and therapeutic resistance. Identifying the genes regulated by Nrf3 is therefore essential for elucidating its physiological functions and involvement in cancer-related pathways. Integrated RNA sequencing and quantitative proteomic analyses have shown that Nrf3 modulates the expression of 176 upregulated and 404 downregulated transcripts, a considerably smaller set compared to Nrf1 and Nrf2 [41]. Many of these targets are functionally linked to extracellular matrix organization and proteasomal activity, while ChIP-based analyses have validated specific cancer-related genes summarized in Table 1. Consistent with these findings, an increasing body of evidence implicates Nrf3 in diverse oncogenic processes, including altered cellular metabolism, enhanced invasion and metastasis, dysregulated cell cycle progression, angiogenesis, therapeutic resistance, and evasion of apoptosis. In colorectal cancer, Nrf3 has even been identified as one of nine potential biomarker genes [42]. Interestingly, its tissue-restricted expression pattern-being highly expressed in the placenta but minimally in most other tissues-may further enhance its potential as a tumor-specific biomarker, since aberrant reactivation in malignant tissues could represent a cancer-specific molecular signature.

**Table 1:** Target genes of Nrf3 and its relation with cancer mechanism

Gene	Validation method	Associated cancer mechanisms	References
DUX4	ChIP-qPCR	Cell cycle regulation and proliferation	[19]
UHMK1	ChIP (unspecified)	Cell cycle regulation and proliferation	[18]
GLUT1	ChIP-seq	Cell metabolism and proliferation	[36]
POMP	ChIP-qPCR	Apoptosis, drug resistance, and proliferation	[48]

Over the past decades, numerous strategies have been employed in cancer research and treatment, including DNA sequencing of human tumor tissues, identification of mutations in oncogenes and signaling pathways, bioinformatic analyses, and the development of cell cycle inhibitors [43]. Recent evidence indicates that Nrf3 plays a critical role in regulating genes involved in cell cycle progression, and its overexpression has been associated with enhanced proliferative capacity in various cancer cell types. Nrf3 transcriptionally represses DUX4, a negative regulator of CDK1, thereby facilitating cell cycle progression and promoting cancer cell proliferation [19]. Moreover, Nrf3 promotes the transcription of UHMK1, a kinase that phosphorylates the CDK inhibitor p27Kip1, thereby modulating cell cycle entry [18].

In addition to its roles in invasion and angiogenesis, Nrf3 has been implicated in cancer metabolism. Tumor cells are known to undergo metabolic reprogramming to meet elevated bioenergetic and biosynthetic demands. A hallmark of this shift is the Warburg effect, characterized by the conversion of glucose to lactate even in the presence of oxygen [44]. Glucose transporter 1 (GLUT1) facilitates glucose uptake across a wide range of cell types and is a central mediator of this metabolic adaptation. Overexpression of Nrf3 in colon cancer cells transcriptionally upregulates GLUT1, thereby enhancing glucose uptake and promoting cell proliferation [36]. These findings suggest that Nrf3 may facilitate metabolic reprogramming by increasing glucose availability in cancer cells. Collectively, these studies highlight the multifaceted oncogenic functions of Nrf3, including its involvement in cell proliferation, metastasis, angiogenesis, and metabolic adaptation. The consistent overexpression of Nrf3 across diverse tumor types and its contribution to tumor progression underscore its potential as a promising therapeutic target in cancer.

In colon cancer cells, siRNA-mediated silencing of Nrf3 leads to downregulation of CCND1 (Cyclin D1) and decreased phosphorylation of pRb1 at Ser807/811, ultimately resulting in G0/G1 cell cycle arrest [45]. Disruption of tumor suppressor pathways represents a fundamental hallmark of cancer progression [46, 47]. In this regard, Nrf3 has been shown to contribute to cell cycle regulation through modulation of TP53- and retinoblastoma (Rb)-associated signaling pathways. Specifically, Nrf3 overexpression enhances the transcription of proteasome maturation protein (POMP), a key component of the 26 S proteasome complex. Elevated levels of POMP promote the ubiquitin-independent degradation of TP53 and Rb, two crucial tumor suppressors. This aberrant degradation leads to reduced apoptotic activity and increased cellular proliferation in colon cancer cells [48]. Moreover, several genes not included in the table but reported to be associated with Nrf3 have also been implicated in critical functions across different cancer types.

**Fig. 3:**
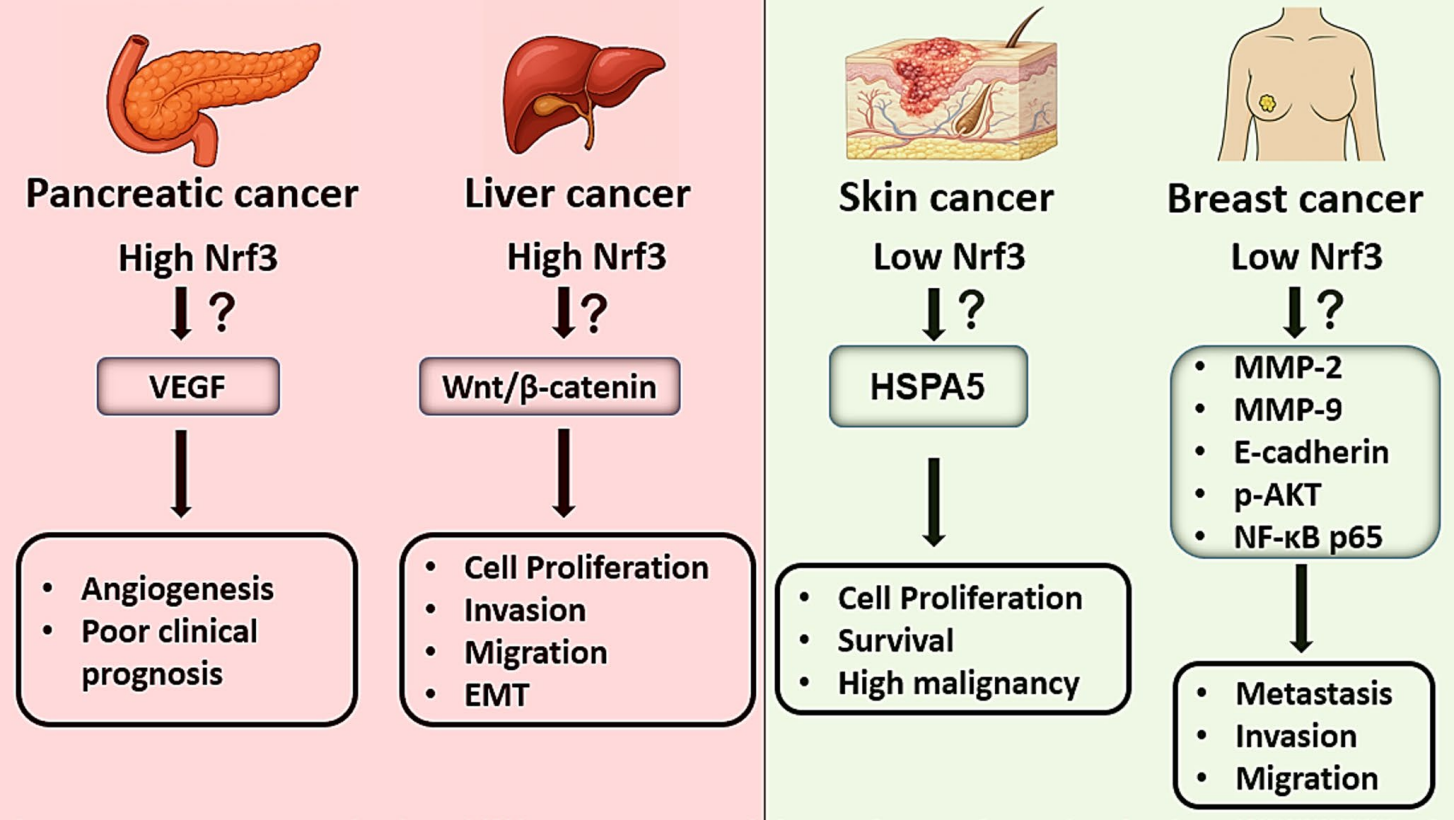
Differential expression and downstream effects of Nrf3 across cancer types

Beyond colorectal cancer, the oncogenic roles of Nrf3 have been investigated in various other malignancies (Fig. 3). In thyroid cancer cells, elevated expression of Nrf3 has been shown to promote tumor cell invasion and metastasis, thereby conferring malignant properties to cancer cells [49]. In hepatocellular carcinoma, Nrf3 expression levels positively correlate with tumor grade, and its silencing via shRNA significantly reduces cellular proliferation, invasion, and migration [50]. Mechanistically, Nrf3 has been reported to activate the Wnt/β-catenin signaling pathway in hepatocellular carcinoma, thereby enhancing cell motility and promoting epithelial-mesenchymal transition (EMT) [51].

In pancreatic cancer, high Nrf3 expression is associated with poor clinical prognosis. Increased Nrf3 levels in patient tumor tissues correlate with elevated expression of vascular endothelial growth factor (VEGF), a key regulator of tumor angiogenesis. Silencing of Nrf3 by siRNA significantly downregulates VEGF expression in pancreatic cancer cells, suggesting that Nrf3 contributes to neovascularization through modulation of pro-angiogenic signaling pathways [52].

Although elevated Nrf3 expression has been reported in various cancer types, its expression appears to be suppressed in specific contexts such as basal and squamous cell carcinoma and breast cancers. Contrary to its mRNA expression profiles, Nrf3 protein levels have been reported to exhibit a marked decrease in actinic keratosis, basal cell carcinoma, and squamous cell carcinoma lesions. These findings suggest that Nrf3 may be a distinctive molecular feature of skin cancer cells exhibiting invasive growth capacity under in vivo conditions [53]. Functional studies conducted in a xenograft mouse model demonstrated that Nrf3 deficiency in tumors derived from SCC13 Nrf3-KO cells is associated with increased proliferative activity. Conversely, Nrf3 overexpression in SCC13 cells did not result in a significant change in tumor growth within the same model. It has been reported that the loss of Nrf3 leads to a marked increase in HSPA5 expression, thereby supporting the survival and migration capacity of SCC cells. Furthermore, pharmacological or genetic inhibition of HSPA5 significantly attenuated the malignant features arising from Nrf3 deficiency, indicating that HSPA5 plays a functional regulatory role in this process.

To evaluate the clinical significance of Nrf3 in breast cancer, immunohistochemical analyses of tumor tissues from 144 patients revealed that Nrf3 expression was absent in the majority of cases (68.7%), while a limited proportion (31.3%) exhibited low expression levels. These findings suggest that Nrf3 expression is generally suppressed in breast cancer and may be associated with a potential tumor-suppressive role [54]. Transfection-induced overexpression of Nrf3 markedly decreased the expression of matrix metalloproteinase-2 (MMP-2) and matrix metalloproteinase-9 (MMP-9), while concomitantly increasing the expression of the epithelial marker E-cadherin. These molecular alterations contributed to the suppression of EMT, thereby resulting in a significant reduction in the migratory and invasive potential of cancer cells. Additionally, exogenous Nrf3 expression inhibited AKT phosphorylation and NF-κB p65 activation, further supporting its inhibitory effect on oncogenic signaling. Collectively, these findings suggest a context-dependent tumor-suppressive function of Nrf3, contrasting with its established oncogenic roles in other cancer types. Moreover, it has been demonstrated that Nrf3 overexpression in TNBC cells specifically suppresses the antioxidant gene NQO1, thereby increasing intracellular ROS levels and subsequently inhibiting the MAPK/ERK signaling pathway. This regulation has been reported to result in the suppression of cell proliferation, migration, and invasion, alongside the EMT process. Additionally, Nrf3-mediated ROS elevation has been shown to trigger HMGB1 release, which induces the polarization of macrophages toward the antitumorigenic M1 phenotype, thereby suppressing tumor progression through immune-mediated mechanisms [55]. Notably, a negative correlation has been observed between DNA methylation and Nrf3 expression in both breast and gastric cancers, and hypermethylation of the Nrf3 gene has been associated with patient survival outcomes [56].

In summary, the role of Nrf3 in cancer biology transcends a conventional oncogenic classification, presenting a highly complex landscape shaped by tissue type and the cellular microenvironment. Primary data derived from colon, thyroid, and pancreatic cancer models suggest that Nrf3 may function as an oncogenic driver by supporting processes such as cell cycle control, metabolic reprogramming, and evasion of apoptosis. In particular, mechanistic frameworks proposed in colorectal cancer-revolving around POMP and GLUT1-provide valuable insights into how this transcription factor might facilitate tumor progression. Conversely, reports indicating that Nrf3 may exhibit tumor-suppressive properties in breast cancer and squamous cell carcinoma (SCC) models clearly underscore the context-dependent nature of its function. While current evidence positions Nrf3 as a promising candidate for biomarker discovery and therapeutic targeting, it remains premature to assert the universality of these transcriptional networks. To fully elucidate this intricate regulation and to clarify the specific clinical contexts in which Nrf3 acts as either an oncogene or a suppressor, more extensive in vivo validations and functional studies are warranted.

### Proteasomal crosstalk of Nrf3 and Nrf1

The proteasome is indispensable for maintaining cellular homeostasis by facilitating the degradation of misfolded or damaged proteins, regulating gene transcription, and modulating immune responses-processes that are largely mediated by the ubiquitin-proteasome system [57]. The 26 S proteasome consists of a 20 S catalytic core particle and two 19 S regulatory particles. The 20 S core is responsible for proteolysis, degrading substrate proteins into peptides approximately 3 to 15 amino acids in length. The 19 S regulatory complexes function as gatekeepers by recognizing polyubiquitinated proteins and enabling their translocation into the 20 S core. Impairment of proteasome function can trigger apoptosis, and cancer cells often exhibit increased proteasomal activity to sustain proliferation and survival. Consequently, proteasome inhibition has been widely investigated as a therapeutic strategy in oncology [40].

Recent studies have highlighted the critical role of Nrf1 in the transcriptional regulation of proteasome subunits. Proteasome inhibition results in the accumulation and activation of Nrf1, which translocates from the ER to the nucleus and binds to AREs, thereby inducing the expression of proteasome-related genes [12]. Similarly, Nrf2, a master regulator of cellular antioxidant defense, contributes to the upregulation of certain proteasome components. In p53-deficient cancer cells, Nrf2 can activate the transcription of proteasome genes through a p53-independent pathway [58]. Additionally, Nrf2 activation under oxidative stress conditions, such as exposure to hydrogen peroxide (H₂O₂), enhances the expression of both the 20 S proteasome and the 11 S proteasome activator complex (PA28αβ) [59].

In addition to Nrf1 and Nrf2, evidence regarding the regulatory role of Nrf3 in proteasome homeostasis is currently limited to a small number of studies performed in specific cancer models. For instance, ChIP analyses in H1299 lung cancer cells have confirmed its direct binding to conserved ARE sequences within the POMP gene, establishing its role in the transcriptional control of this essential assembly chaperone within this specific cellular context [48, 60]. Furthermore, DNA microarray analysis conducted in HCT116 colorectal cancer cells identified cytoplasmic polyadenylation element-binding protein 3 (CPEB3) as one of the 146 genes positively associated with Nrf3 expression. In this model, CPEB3 inhibits the translation of Nrf1 at ER-associated polysomes, suggesting that Nrf3 overexpression indirectly suppresses Nrf1 protein levels. Notably, while Nrf1 knockdown alone may not significantly reduce proteasome gene expression in these cells, simultaneous CPEB3 overexpression and Nrf3 depletion results in decreased proteasome expression [61]. These findings suggest that Nrf3 may serve as a specialized or compensatory regulator of proteasome components in certain malignancies or under conditions where Nrf1 function is compromised. While Nrf1 remains the established primary activator of proteasome genes, this potentially distinct role of Nrf3 requires further research to determine whether it contributes to proteasome inhibitor resistance across different malignancies or remains limited to specific cellular models.

### Nrf3 in redox homeostasis and antioxidant control

ROS are critical modulators of cellular functions, influencing a wide range of physiological processes such as cell survival, stress responses, and inflammation. While ROS are predominantly generated endogenously as byproducts of mitochondrial respiration and various metabolic pathways, they may also originate from a variety of exogenous sources, including cigarette smoke, pharmaceutical agents, pathogenic infections, hyperoxia, ionizing radiation, and heavy metal exposure [62]. When ROS levels exceed the cell’s antioxidant capacity, a state of oxidative stress occurs. Accumulation of ROS can lead to extensive damage to macromolecules-including carbohydrates, lipids, proteins, and nucleic acids-and plays a significant role in carcinogenesis by promoting genomic instability and mutation accumulation [63–66].

To defend against oxidative damage, cells activate complex antioxidant mechanisms that are primarily regulated through AREs at the transcriptional level. Nrf2 is widely recognized as the central transcriptional regulator of oxidative stress responses. Upon ROS stimulation, Nrf2 translocates into the nucleus, dimerizes with small Maf proteins, binds to ARE sequences, and induces the transcription of numerous antioxidant and cytoprotective genes [67, 68]. For example, in H₂O₂-treated lung cancer cells, Nrf2 enhances the transcription of the antioxidant enzyme Prdx6 by binding to ARE sites within its promoter. Notably, the same study demonstrated that increased expression of Nrf3 suppressed Prdx6 expression in a dose-dependent manner, suggesting an opposing regulatory effect [69].

Supporting this role, other studies indicate that Nrf3 may act as a negative regulator of ARE-mediated gene expression. In hepatocellular carcinoma cells treated with tert-butylhydroquinone (t-BHQ), overexpression of Nrf3 was shown to inhibit the expression of NQO1, a canonical Nrf2 target gene [70]. In line with these findings, Pepe et al. reported elevated Nrf3 expression during smooth muscle cell differentiation. In embryonic stem cells, forced Nrf3 expression downregulated antioxidant proteins such as NQO1 and Prdx, while simultaneously increasing intracellular ROS levels. This effect was associated with the upregulation of Nox4, a key enzymatic source of ROS in mammalian cells [27]. A study by Xing (2024) confirmed that Nrf3 overexpression in breast cancer inhibits the expression of the antioxidant enzyme NQO1 through a direct transcriptional mechanism.

Collectively, these findings underscore the pivotal role of Nrf3 in modulating redox homeostasis and cellular differentiation. By promoting ROS generation and suppressing ARE-dependent antioxidant gene expression, Nrf3 appears to influence lineage specification and tumor cell behavior. Notably, current literature identifies Nrf3 as a pro-oxidant tumor suppressor, specifically in Triple-Negative Breast Cancer (TNBC). Beyond its broad role in developmental biology, Nrf3 has been shown to directly repress the transcription of antioxidant genes like NQO1, leading to a targeted intracellular ROS accumulation. Unlike physiological ROS signaling, this Nrf3-induced oxidative surge effectively arrests oncogenic progression by inhibiting the MAPK/ERK signaling [55, 71].

## MicroRNAs targeting Nrf3: dual roles in tumor suppression and oncogenesis

MicroRNAs (miRNAs) are a class of small, non-coding RNAs approximately 22 nucleotides in length that regulate gene expression post-transcriptionally by binding to complementary sequences within target mRNAs. Through this mechanism, miRNAs can function either as oncogenes or tumor suppressors and are involved in a variety of biological processes, including cell proliferation, differentiation, apoptosis, and carcinogenesis [72]. Dysregulation of miRNA expression has been widely implicated in cancer initiation, progression, and malignancy across various tumor types [73].

Nrf3 is frequently overexpressed in cancer cells, suggesting a potential oncogenic role. Given this observation, investigating the expression profiles of miRNAs that regulate Nrf3 may offer novel insights into its functional role in tumorigenesis. For instance, in colon adenocarcinoma, miR-23b-3p has been identified as a direct post-transcriptional regulator of Nrf3. Expression analyses have revealed that miR-23b-3p is significantly downregulated in both colon adenocarcinoma tissues and cell lines, while its overexpression suppresses tumor malignancy in both in vitro and in vivo models [74].

Another miRNA of interest is miR-1246, which has been shown to exhibit oncogenic properties in various cancer types. It is markedly upregulated in breast cancer and negatively correlates with patient survival. Nrf3 has been identified as a downstream target of miR-1246 in this context [28]. Interestingly, although Nrf3 is generally associated with tumor progression, some studies have reported tumor-suppressive effects of Nrf3 in breast cancer [54]. Notably, inhibition of miR-1246 in breast cancer cells leads to increased Nrf3 expression, which in turn is associated with reduced phosphorylation levels of AKT, ERK, and NF-κB-three major components of pro-survival signaling pathways [28].

Additional miRNAs predicted to regulate Nrf3 are listed in Table 2. Among the 247 predicted target genes of miR-99a-3p in head and neck squamous cell carcinoma (HNSC), Nrf3 has been identified as a candidate. miR-99a-3p, which exerts tumor-suppressive effects, is frequently downregulated in HNSC and its reduced expression is associated with poor prognosis [75, 76]. Consistently, data from Gene Expression Profiling Interactive Analysis 2 (GEPIA2) indicate that Nrf3 expression is significantly elevated in HNSC tissues compared to normal controls (http://gepia2.cancer-pku.cn/#general). These findings suggest that miR-99a-3p may serve as a potential therapeutic candidate for targeting Nrf3 in HNSC.

**Table 2:** Possible miRNAs targeting Nrf3

Cancer	miRNA	Function	Status / Evidence	References
Head and neck squamous cell carcinoma	miR-99a-3p	Tumor-suppressive	In silico analysis	[75]
Pancreatic ductal adenocarcinoma	miR-30c-5p	Tumor-suppressive	In silico analysis	[77]
	miR-30c-2-3p		In silico analysis	
Breast cancer	miR-26a	Tumor-suppressive	In silico analysis	[80]
	miR-1246	Oncogenic	In vitro and in vivo validated	[29]
Colorectal adenocarcinoma	miR-23b-3p	Tumor-suppressive	In vitro and in vivo validated	[74]

In pancreatic ductal adenocarcinoma (PDAC), the expression of miR-30c-5p and miR-30c-2-3p is markedly reduced in tumor tissues compared to normal pancreatic tissues. Both miRNAs are known to exhibit tumor-suppressive functions and have been predicted to target several oncogenes, including Nrf3, which is ranked among the top ten most relevant targets [77]. As previously discussed, Nrf3 is significantly upregulated in PDAC and contributes to oncogenic signaling pathways [52]. These results suggest that miR-30c-5p and miR-30c-2-3p may modulate Nrf3 expression in pancreatic cancer, and that their downregulation may contribute to malignancy via this regulatory axis.

Moreover, p53 has been shown to regulate cell cycle progression and apoptosis, in part, through transcriptional control of multiple miRNAs [78, 79]. Literature-based bioinformatic analyses suggest that miR-26a, one of the p53-regulated miRNAs, targets Nrf3. It has been proposed that p53 may transcriptionally upregulate miR-26a in breast cancer, potentially linking the p53/miR-26a/Nrf3 axis to tumor suppression [79, 80].

## Nrf3-mediated lipid homeostasis

In the preceding sections, the structural characteristics and regulatory mechanisms of the transcription factor Nrf3 were discussed in the context of its roles in antioxidant defense, inflammation, and oncogenesis. Beyond these functions, accumulating evidence highlights a critical role for Nrf3 in maintaining protein and lipid homeostasis. By modulating key metabolic pathways, Nrf3 influences vital physiological processes such as cellular survival and proliferation. Aberrant regulation of Nrf3 has been implicated in the pathogenesis of diverse diseases, including cancer, neurodegenerative disorders, metabolic syndromes, and obesity [81].

Lipids such as cholesterol and fatty acids are fundamental not only for energy storage and membrane synthesis but also for regulating intracellular signaling pathways. Central to lipid metabolic control are sterol regulatory element-binding proteins (SREBPs), a family of membrane-bound transcription factors. Under conditions of intracellular cholesterol depletion, SREBPs are mobilized from the ER to the Golgi apparatus, where site-specific proteolytic cleavage liberates their N-terminal active fragments. These fragments subsequently translocate to the nucleus to activate transcription of lipid-related genes. Among the two major isoforms, SREBP1 primarily governs fatty acid synthesis and adipogenic differentiation, whereas SREBP2 controls the transcription of genes involved in cholesterol biosynthesis [82].

Recent studies have revealed that Nrf3 exerts regulatory control over lipid metabolism through multiple mechanisms. Notably, Nrf3 modulates the mevalonate pathway via SREBP2 and regulates cholesterol uptake through the geranylgeranyl pyrophosphate (GGPP)-dependent activity of geranylgeranyl diphosphate synthase 1 (GGPS1). It also influences the function of small GTPases such as RAB5, which are linked to Ras-mediated signaling cascades [83]. Specifically, Nrf3 promotes the transcription of canonical SREBP2 target genes including HMGCS1 (3-hydroxy-3-methylglutaryl-CoA synthase 1) and HMGCR (3-hydroxy-3-methylglutaryl-CoA reductase), the latter being the rate-limiting enzyme in cholesterol biosynthesis from acetyl-CoA via the mevalonate pathway [84]. ChIP analyses by Landt et al. (2012) confirmed direct Nrf3 binding to AREs located in the promoter regions of SREBP2 and HMGCR, supporting its transcriptional activation role in enhancing mevalonate pathway flux [85]. Interestingly, despite the upregulation of HMGCR expression and enzymatic activity, intracellular cholesterol levels remain largely unchanged following Nrf3 activation.

In addition to its effects on cholesterol metabolism, Nrf3 regulates intracellular lipid balance by inducing the expression of GGPS1, which catalyzes the conversion of farnesyl pyrophosphate to lanosterol and GGPP. The accumulation of GGPP suppresses fatty acid biosynthesis and limits intracellular lipid storage by negatively modulating SREBP1 activity [86]. Supporting this mechanism, transcriptomic analyses have demonstrated an inverse correlation between Nrf3 expression and genes involved in fatty acid metabolism [83].

The small GTPase RAB5 functions as a pivotal mediator of early endocytosis and macropinocytosis-a process facilitating bulk fluid-phase nutrient uptake. Proper subcellular localization and membrane association of RAB5 require post-translational prenylation, a modification dependent on GGPP availability. Nrf3 enhances prenylation and activity of RAB5, thereby promoting RAB5-dependent endocytic trafficking through GGPP production [87]. Additionally, Nrf3 has been shown to stimulate macropinocytosis, and elevated activity of this pathway has been observed in the intestinal tissues of Nrf3-transgenic mice [83]. Given the central role of the intestine in systemic cholesterol absorption, dysregulation of this pathway may contribute to aberrant lipid uptake and obesity. Since obesity is a well-established risk factor for colorectal cancer, these findings suggest a mechanistic link between Nrf3-mediated lipid homeostasis, intestinal absorption, and tumorigenesis [88]. However, it should be noted that the current understanding of the Nrf3-SREBP2-macropinocytosis axis is primarily derived from animal models and correlational molecular analyses. While these preclinical data provide a compelling framework, the direct causal involvement of this pathway in human colorectal cancer remains to be fully elucidated. Extensive clinical and epidemiological studies are required to validate whether these Nrf3-driven metabolic shifts translate into measurable risks in human populations and to confirm their potential as therapeutic targets.

## Conclusions and future perspectives

The findings summarized in this review reveal that Nrf3 is not only the “youngest” member of the CNC bZIP family but also a multifaceted regulator of cellular homeostasis. Its ER-dependent maturation process, the proteolytic cleavages mediated by DDI2 or potentially NGLY1, the degradation cycle along the ERAD/SCF axis, and the stress-sensitive translocation steps remain incompletely mapped. Filling these biochemical gaps will be critical for understanding the tissue-specific functions of Nrf3. Evidence indicates that Nrf3 can exert a dual role in cancer: on the one hand, promoting proliferation, metabolic reprogramming, angiogenesis, and invasion through targets such as POMP, GLUT1, SREBP2-mevalonate, and VEGF; on the other hand, inhibiting oncogenic MAPK/ERK and AKT signaling axes, thereby reducing metastatic capacity and EMT in specific settings, such as certain breast and squamous cell carcinoma models. This “two-faced” effect likely intersects with tumor type-specific epigenetic landscapes, miRNA networks (e.g., miR-23b-3p, miR-99a-3p, miR-1246), and microenvironmental factors such as cytokine-Wnt-NF-κB signaling. Future studies should address (i) the promoter motifs to which Nrf3 displays high affinity in place of AREs, (ii) the mechanisms by which it regulates extracellular lipid uptake and macropinocytosis, and (iii) how it integrates ROS accumulation into differentiation decisions-approaches that will require advanced omics platforms and live-cell imaging models. Moreover, combination strategies involving small-molecule inhibitors targeting Nrf3, proteasome inhibitors, or mevalonate pathway blockers represent promising candidates for clinical translation in tumors with high therapeutic susceptibility, particularly pancreatic, hepatocellular, and thyroid cancers.

## Data Availability

No datasets were generated or analysed during the current study.
